# Supplementary material for: The interactome of intact mitochondria by cross-linking mass spectrometry provides evidence for coexisting respiratory supercomplexes
Source: Mol Cell Proteomics. 2017 Dec 8;17(2):216–32. doi: 10.1074/mcp.RA117.000470 (PMC5795388; doi:10.1074/mcp.RA117.000470)
Supplement: Supplemental Data [file supp_RA117.000470_133922_0_supp_23978_fzffwf.zip › spectra_annotation/mito_DR_spectra_annotation/67-1-2-1-4-1.pdf]

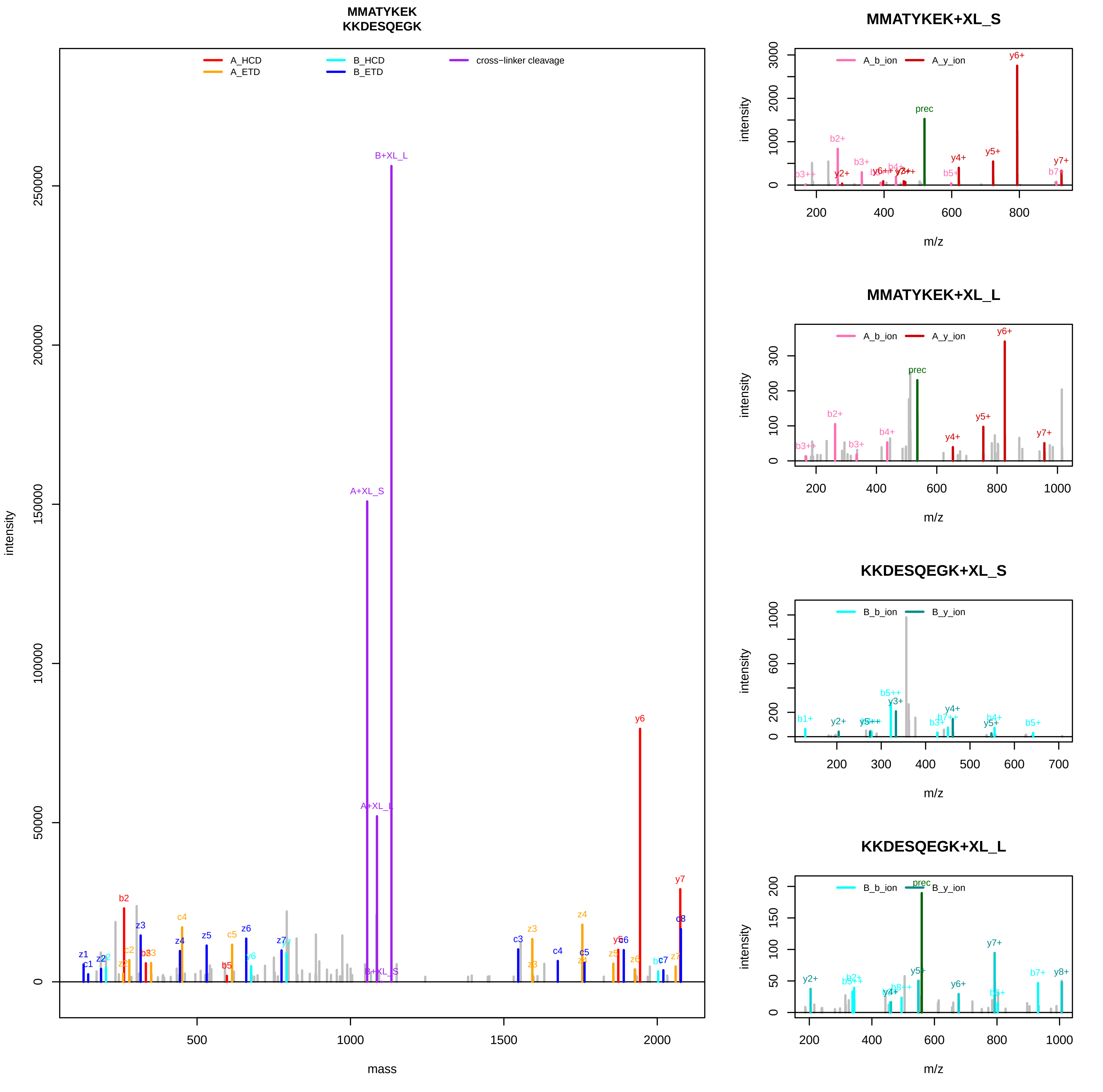

MMATYKEK
KKDESQEGK
A_HCD
A_ETD
B_HCD
B_ETD
cross−linker cleavage
B+XL_L
A+XL_S
A+XL_L
B+XL_S
y6
y7
b2
c8
z4
intensity
mass
0
50000
100000
150000
200000
250000
500
1000
1500
2000
MMATYKEK+XL_S
A_b_ion
A_y_ion
prec
y6+
y5+
y4+
b2+
y7+
m/z
MMATYKEK+XL_L
A_b_ion
A_y_ion
prec
y6+
y5+
y7+
y4+
b2+
b4+
b3+
m/z
KKDESQEGK+XL_S
B_b_ion
B_y_ion
b5++
y3+
y4+
b1+
y2+
b5+
m/z
KKDESQEGK+XL_L
B_b_ion
B_y_ion
prec
y7+
y2+
y5+
y6+
b7+
y8+
m/z
